# Early Surgery versus Initial Conservative Treatment in Patients with Traumatic Intracerebral Hemorrhage (STITCH[Trauma]): The First Randomized Trial

**DOI:** 10.1089/neu.2014.3644

**Published:** 2015-09-01

**Authors:** A. David Mendelow, Barbara A. Gregson, Elise N. Rowan, Richard Francis, Elaine McColl, Paul McNamee, Iain R. Chambers, Andreas Unterberg, Dwayne Boyers, Patrick M. Mitchell

**Affiliations:** ^1^ Neurosurgical Trials Group, Newcastle University, Newcastle upon Tyne, United Kingdom.; ^2^ Newcastle Clinical Trials Unit, Newcastle University, Newcastle upon Tyne, United Kingdom.; ^3^ Health Economics Research Unit, University of Aberdeen, Aberdeen, United Kingdom.; ^4^ Department of Medical Physics, South Tees Hospitals Foundation Trust, James Cook University Hospital, Middlesbrough, United Kingdom.; ^5^ Department of Neurosurgery, Heidelberg University, Heidelberg, Germany.; ^6^ Royal Victoria Infirmary, Newcastle upon Tyne, United Kingdom.

**Keywords:** craniotomy, intracerebral hemorrhage, randomized, controlled trial, traumatic brain injury

## Abstract

Intraparenchymal hemorrhages occur in a proportion of severe traumatic brain injury TBI patients, but the role of surgery in their treatment is unclear. This international multi-center, patient-randomized, parallel-group trial compared early surgery (hematoma evacuation within 12 h of randomization) with initial conservative treatment (subsequent evacuation allowed if deemed necessary). Patients were randomized using an independent randomization service within 48 h of TBI. Patients were eligible if they had no more than two intraparenchymal hemorrhages of 10 mL or more and did not have an extradural or subdural hematoma that required surgery. The primary outcome measure was the traditional dichotomous split of the Glasgow Outcome Scale obtained by postal questionnaires sent directly to patients at 6 months. The trial was halted early by the UK funding agency (NIHR HTA) for failure to recruit sufficient patients from the UK (trial registration: ISRCTN19321911). A total of 170 patients were randomized from 31 of 59 registered centers worldwide. Of 82 patients randomized to early surgery with complete follow-up, 30 (37%) had an unfavorable outcome. Of 85 patients randomized to initial conservative treatment with complete follow-up, 40 (47%) had an unfavorable outcome (odds ratio, 0.65; 95% confidence interval, CI 0.35, 1.21; *p*=0.17), with an absolute benefit of 10.5% (CI, −4.4–25.3%). There were significantly more deaths in the first 6 months in the initial conservative treatment group (33% vs. 15%; *p*=0.006). The 10.5% absolute benefit with early surgery was consistent with the initial power calculation. However, with the low sample size resulting from the premature termination, we cannot exclude the possibility that this could be a chance finding. A further trial is required urgently to assess whether this encouraging signal can be confirmed.

## Introduction

In the UK, there are 1.4 million presentations of traumatic brain injury (TBI) at emergency departments each year.^1^ The incidence worldwide varies between 56 and 430 per 100,000 population each year,^2^ with the highest incidence being in Asia (344 per 100,000) and the lowest in the United States (103 per 100,000).^3^ The mortality rate for severe isolated TBI in the UK varied between 16% and 40%,^4^ which is similar to the world-wide rates that vary between 15% and 38%.^3^ Intracranial hemorrhage occurs in more than 60% of serious TBIs in one or more of three types: extradural, subdural, and intraparenchymal. Prompt surgical removal of significant subdural (SDH) and extradural hemorrhage (EDH) is well established and widely accepted. Intraparenchymal hemorrhage is more common than both these other types and is clearly associated with a worse outcome, but the role for surgery and its timing remains undefined. Several terms are used to describe the condition, including traumatic intraparenchymal hemorrhage, traumatic intracerebral hemorrhage (TICH), and contusion.

Traditional neurosurgical management of patients with severe TBI (sTBI) is frequently based on intracranial pressure (ICP) measurement. Patients with high ICP (>30 mm Hg) would typically undergo craniotomy and those with low ICP (<20 mm Hg) would be managed conservatively. Patients with ICP between 20 and 30 mm Hg would be observed closely and undergo craniotomy if the ICP rises.^5^ This ICP-based approach has been recommended by the Brain Trauma Foundation,^6^ although publication of the BEST TRIP trial from Latin America^7^ has engendered further debate. However, not all hospitals have or use ICP monitoring for patients with TICH, even though they may be classified as having an sTBI. Early management of patients with TICH requires evaluation to determine whether early surgery should become part of the standard of care in the same way it is for significant EDHs^8^ and SDHs.^9^

The aim of early surgical TICH removal is to prevent secondary brain injury, which is thought to be caused by a number of mechanisms. Extravasated blood is believed to be neurotoxic, leading to secondary injury that may be avoided by early surgical removal. Larger TICHs may be associated with an ischemic penumbra of brain tissue that could be salvaged and some TICHs expand to the point where they cause mass effect, resulting in secondary brain injury. Contused brain does not seem to recover and appears later as encephalomalacic brain tissue loss on convalescent phase imaging. Removal of irreversibly damaged brain contusion with TICH does not increase tissue loss. As with spontaneous intracerebral hemorrhage (SICH), there are patients who will deteriorate clinically and the question of early surgery arises to anticipate such secondary damage.

Use of surgery for TICH varies around the world. It is more frequent in Asia than in Europe or North America. There have been randomized trials of surgery for SICH,^10,11^ but none thus far of surgery for TICH. Patients suffering a TICH tend to be younger than those suffering a SICH, and therefore level of disability may have a larger effect on ability to return to employment and economic output. TICHs are more likely to be lobar, superficial, and have a medium-sized volume (25–65 cc).^12^ These differences between the conditions mean that the role of surgery for TICH cannot be directly derived from results of the published trials of surgery for SICH. If early surgery is of benefit to TICH patients, then implementation of early referral and diagnosis with immediate treatment may reduce incidence of death and disability in this specific group of TBI patients.

The National Institute for Health and Care Excellence (NICE) in the UK published their second edition of guidelines for the triage and management of TBI patients in 2007,^13^ and the Brain Trauma Foundation published their guidelines for the surgical management of TBI in 2006.^14^ Both of these organizations have emphasized that studies have been observational and that there is a lack of class 1 evidence from well-designed randomized, controlled trials. Those unrandomized studies that attempt to compare outcome between surgical and nonsurgical groups cannot adequately control for known prognostic variables. The NICE recommended in the 2007 guideline that research is needed to develop a consensus on criteria for lesions not currently considered to be surgically significant, namely, TICH. This recommendation facilitated the funding of [STITCH(TRAUMA)] to find out whether early surgery would improve outcomes, compared with initial conservative treatment, in patients with supratentorial TICH.

## Methods

### Trial design and participants

The STITCH(Trauma) protocol has been published.^15^ This was an international, multicenter, prospective, patient-randomized, parallel-group pragmatic trial comparing early surgical evacuation of TICH with initial conservative treatment (ISRCTN 19321911, UK NIHR-HTA grant no.: 07/37/16). Ethical committee favorable opinion was obtained from the Southampton Multicenter Research Ethics Committee (REF: 09/H0502/68, June 15, 2009). Local ethical approval was obtained for each participating center. The trial was conducted according to Medical Research Council good clinical practice guidelines. Formal agreements were in place between the sponsor (Newcastle upon Tyne NHS Hospitals Foundation Trust), the holder of the study funding (Newcastle University, Newcastle upon Tyne, UK), and each participating hospital before commencing the study at each site.

Only TICH patients for whom the treating neurosurgeon was in equipoise about the benefits of early surgical evacuation, compared with initial conservative treatment, were eligible for the trial. Patients considered for the trial had had a computed tomography (CT) scan to confirm the diagnosis and size as well as location of the hematoma. Clotting or coagulation problems were corrected before randomization as per local standard clinical practice. Patients were included if they were adults within 48 h of TBI and had evidence of a TICH on CT with a confluent volume of attenuation significantly raised above that of the background white and gray matter greater than 10 mL calculated by: (length×width×height)/2 in cm.^16^ (Initially, the time criterion was within 24 h of TBI, but subsequent to an investigators' meeting this was increased to allow time for patients to reach neurosurgery and for the TICH to develop.)

Exclusion criteria were: a significant surface hematoma (EDH or SDH) requiring surgery; three or more separate hematomas fulfilling the inclusion criteria; a cerebellar hemorrhage/contusion; surgery could not be performed within 12 h of randomization; severe pre-existing physical or mental disability or comorbidity that would lead to a poor outcome even if the patient made a full recovery from the TBI; permanent residence outside a study country preventing follow-up; and if the patient and/or relative expressed a strong preference for one treatment modality.

Written witnessed informed consent of the patients or their relatives was obtained by neurosurgical staff before randomization.

In total, 59 neurosurgical units in 20 countries completed all regulatory requirements and registered with the trial.

### Interventions

The two trial interventions were early surgery or initial conservative treatment. Early surgery was early evacuation of the hematoma by a method of the surgeon's choice (within 12 h of randomization), combined with appropriate best medical treatment. Initial conservative treatment was best medical treatment combined with delayed (more than 12 h after randomisation) evacuation if it became appropriate later. Both groups were monitored according to local standard neurosurgical practice.

Best medical treatment could include (depending on the practices within the center) monitoring of ICP or other modalities and management of metabolism, sodium osmotic pressure, temperature, and blood gasses.

All patients had a CT scan at 5 days (±2 days) after randomization to assess changes in hematoma size with and without surgery.

Information was collected about the status (Glasgow Coma Score [GCS] and focal signs) of patients through the first 5 days of their trial progress as well as ICP/CPP (cerebral perfusion pressure) measures (in invasively monitored patients), to describe any change in status that led to a change in equipoise for the treating neurosurgeon, and subsequent surgery in patients initially randomized to conservative treatment. At 2 weeks after randomization or at discharge or death (whichever occurred first), a discharge/2-week form was completed by the responsible neurosurgeon or research nurse. This form recorded the patient's status at that time, the mechanism of injury, whether and when they had surgery (including why, if randomized to initial conservative treatment, or why not, if randomized to early surgery), the GCS and localizing features for the 5 days after randomisation, the occurrence of any adverse events (AEs) after randomization (including death, pulmonary embolism, deep vein thrombosis, and surgical site infection), and past medical history.

Before assessing outcome each patient's GP (in the UK) or consultant (outside the UK) was contacted to check that the patient was alive, confirm the patient's place of residence, and complete a major AEs form.

### Randomization and masking

To minimise biases resulting from lack of concealment of allocation, randomization was undertaken centrally by an independent 24-h telephone and Web randomization service based in Aberdeen University (Aberdeen, UK). Allocation was stratified by geographical region, with a minimization algorithm based on age group, and severity (as measured by whether or not the pupils were equal and reacting), with a random component (i.e., with probability of 80%).

Throughout the study, data broken down by treatment assignment were never provided to individual investigators or to the study team. Outcome was assessed by questionnaires to patients. If it was necessary to administer these questionnaires in person, then the interviewer was blind to treatment allocation. Outcome assignment, data cleaning, and CT assessment were all made before unblinding the treatment assignment.

### Outcomes

Outcome was assessed at 6 months by a postal questionnaire translated into the appropriate languages and mailed to patients or their relatives/carers. In those centers where the postal systems were problematic, or there were literacy or language problems, questionnaires could be completed by a social worker or research nurse, who did not know the treatment allocation, in interview with the patient or relative.

Primary outcome was based on the 6-month Glasgow Outcome Scale (GOS) dichotomized into favorable and unfavorable outcome; dead, vegetative, and severe disability were coded as unfavorable and moderate disability and good recovery as favorable. Secondary outcomes were mortality, time to death, extended Glasgow Outcome Scale (GOSE), Rankin, and European Quality of Life Five Dimension Scale (EQ-5D) at 6 months. Crossover and major event rates (death, pulmonary embolism or deep vein thrombosis, infection, and rehemorrhage) in each treatment group were also reported.

### Statistical analysis

Previous observational studies have suggested a favorable outcome in the nonoperated group of approximately 40% and a favorable outcome in the surgical group of approximately 60–70%. Target sample size was 840 (420 in each arm). This was calculated assuming a more conservative favorable outcome (good recovery or moderate disability on the GOS) of 50% from conservative treatment, 10% benefit (i.e., 50% vs. 60%) from surgery, 5% significance with 80% power, and a safety margin built in to allow for loss to follow-up, making the total target sample size 840 patients (420 randomized to each treatment arm).

The independent data monitoring committee (DMC) reviewed data from the study after 50, 100, and 150 patients had been recruited. These interim reviews were confidential to only the data manager and the DMC. The trial was only to be stopped early by the DMC if one or other treatment policy showed an advantage at a very high significance level, or if recruitment rates were unexpectedly low. The DMC recommended, at each review, that the trial should continue. However, in February 2012, the funding agency decided to halt this international study with effect from the end of September 2012 for “failure to recruit in the UK.” At this time, 6 patients had been recruited from the UK, despite our actively encouraging UK center participation throughout the trial. In total, 10 of the possible 36 UK centers expressed written interest in the study, but only seven completed all regulatory requirements to join the trial. Considerable effort was expended in trying continuously to recruit UK centers and raise the profile of the study—the study team had a high profile at the national neurosurgical meetings and national neurosurgical research meetings giving presentations about progress on the study and manning a stand where investigators could discuss the study with the team. Monthly e-mail newsletters were sent to neurosurgical departments and paper copies were posted quarterly. Talks were given in individual centers to encourage recruitment. Help with all regulatory submissions was also provided. Despite these considerable efforts, only 6 patients could be recruited in the UK.

Analysis was on an intention-to-treat (ITT) basis. The analysis plan was adapted, following the decision from the funding agency to halt the trial early, agreed by the trial steering committee and published on the study website before unblinding the data. The primary analysis was a simple categorical frequency comparison using the uncorrected chi-squared test for favorable and unfavorable outcomes at 6 months.^17,18^ A sensitivity analysis using logistic regression was undertaken to adjust for age, volume of hematoma, and GCS.

Secondary outcome analyses included proportional odds model analysis of GOS, GOSE, and Rankin at 6 months, Kaplan-Meier's survival curve with log-rank test, and mortality. For dichotomized outcomes, absolute differences and 95% confidence intervals (CIs) were reported.

Minimal subgroup analysis was undertaken and regarded as exploratory. Odds ratios (ORs) and 95% CIs were reported for the following subgroups: age (two bands using randomization strata: <50, 50, or more—given that there were very few patients over 70 years of age, the two upper age bands were combined); volume of hematoma (using median split <=23 and >23 mL); GCS (using standard classification of TBI severe, moderate, or minor: 3–8, 9–12, or 13–15); time from ictus to randomization (using median split <21 and >=21 h); and geographical region (four bands: Europe, India, China, and other). Interaction tests (chi-squared test for heterogeneity) were undertaken and relevant *p* values reported.

### Costing analysis

The costing analysis was undertaken on the ITT basis from an international health services perspective. Resource-use requirements to deliver the interventions (e.g., staff time and overheads) and time spent on hospital wards were collected using site-specific questionnaires and case report forms. Hospital readmissions were reported on participant outcome questionnaires. Costing followed recommended procedures for international studies,^19,20^ applying country-specific unit costs (sourced from site-specific questionnaires) to resource-use data to generate total costs. Costs were transformed into 2013 international dollars^21^ and reported as mean (standard deviation; SD) for each treatment group.

A generalized linear regression model (GLM), specifying a gamma family and identity link, was used to estimate the impact of treatment allocation on costs accounting for skewed data and adjusting for patient characteristics (age and sex). Sensitivity analyses explored the use of alternative models for analyzing costs. Subgroup analysis presented raw mean costs according to World Bank classifications,^22^ based on gross national income (GNI) as follows: low income (GNI <=$1,005, e.g., Nepal); lower middle income (GNI $1,006–$3,975, e.g., India); upper middle income (GNI $3,976–$12,275, e.g., China); and high income (GNI >=f $12,276, e.g., western Europe and United States). Owing to small sample sizes, regression analyses were not undertaken.

### Role of the funding source

Neither the sponsor nor the funder of the study had any role in study design, data gathering, analysis and interpretation, or writing the report. The corresponding author, E.N.R., and R.F. had full access to all the data in the study, and all members of the writing committee had responsibility for the decision to submit.

## Results

**FIG. 1.**
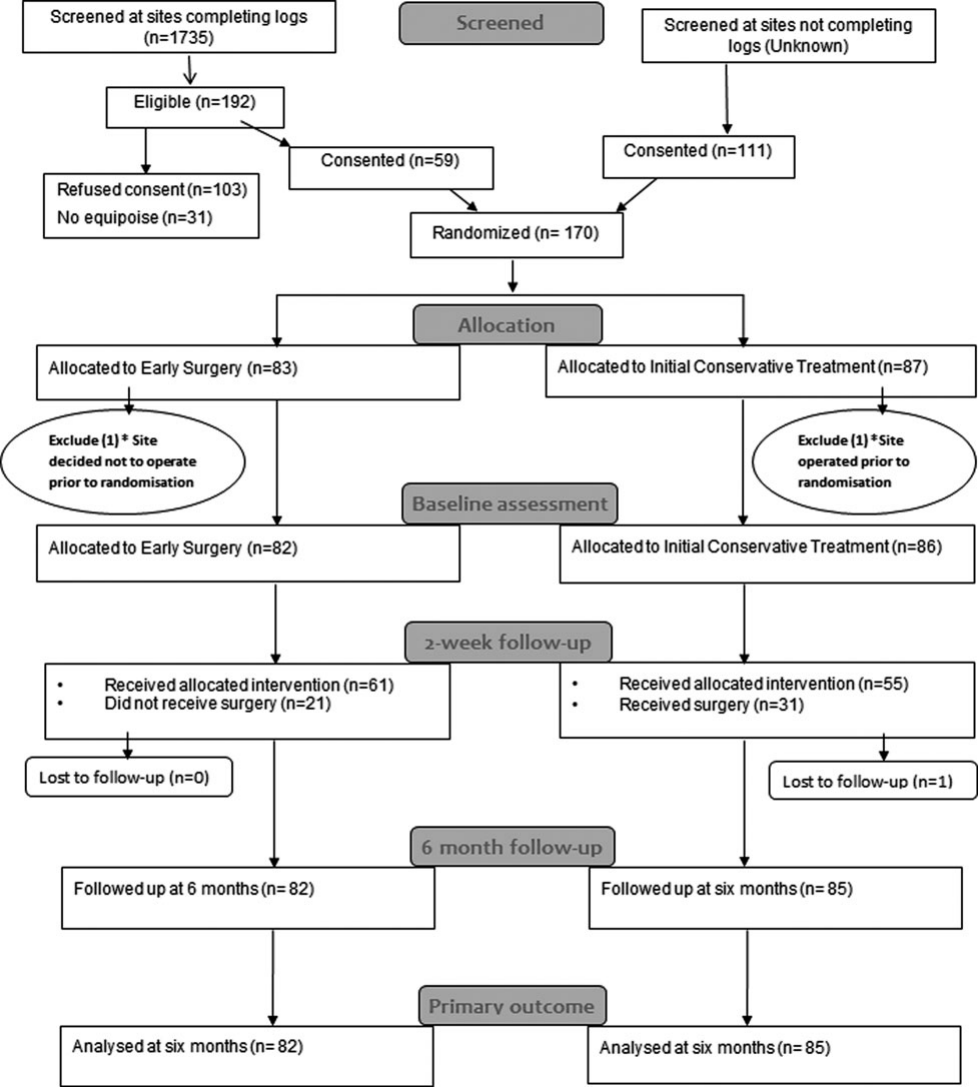
Flow chart for STITCH(Trauma) patients. *One site recruited 1 patient, but had undertaken surgery before randomization—the patient was allocated to initial conservative treatment; another site recruited 1 patient, for whom a treatment decision not to operate was made before the patient was randomized—this patient was allocated to early surgery. Because of the severe breach of protocol, these patients were excluded.

One hundred and seventy patients were recruited from 31 centers in 13 countries between December 2009 and September 2012 and randomly assigned to treatment groups: 83 to early surgery and 87 to initial conservative treatment. Two patients were excluded because the treatment decision was made before randomization: In one case, the patient had surgery before randomization and in the other an early decision was made not to operate (Fig. 1). These were serious protocol violations. All other patients were included in the analysis, which therefore reports results for 82 patients assigned to early surgery and 86 assigned to initial conservative treatment.

Table 1 shows the distribution of baseline variables between the two treatment groups. Patients ranged in age from 16 to 83, with a median age of 50, and 122 (73%) were male. Before the TBI, 164 (98%) were Rankin 0 or 1, and 22 (13%) had a history of cardiovascular disease. The main causes of the TBI were road traffic accidents (RTAs; 113; 67%) and falls (47; 28%). Most of the RTAs were motorbike riders (45; 40%) or pedestrians (29; 26%). Sixty-eight patients (40%) were admitted to another hospital before their transfer to the neurosurgical unit. At the time of randomization, 70 (42%) patients had a GCS of 13–15, 71 (42%) a GCS of 9–12, and 27 (16%) a GCS of 8 or less. The volume of the largest hematoma varied between 10 and 97 mL, with a median of 23 mL, and 61 (36%) patients had a second hematoma between 0 and 26 mL, with median of 3 mL. There were no differences in the three individual components of the GCS, handedness, or characteristics of the second hematoma.

**Table 1.** Baseline Variables

*Variable*	*Early surgery (*N*=82)*	*Initial conservative treatment (*N*=86)*
Age (years),		
median (IQR) range	51 (32–63) 18–83	50 (33–61) 16–77
Mean (SD)	48 (17.7)	48 (16.9)
Age band (%)		
<50	37 (45)	42 (49)
50–69	34 (42)	33 (38)
70+	11 (13)	11 (13)
Sex (%)		
Male	57 (70)	65 (76)
Female	25 (30)	21 (24)
GCS total (%)		
3	0 (0)	1 (1)
4	0 (0)	0 (0)
5	1 (1)	2 (2)
6	6 (7)	3 (3)
7	4 (5)	3 (3)
8	1 (1)	6 (7)
9	11 (13)	8 (9)
10	11 (13)	14 (16)
11	6 (7)	8 (9)
12	6 (7)	7 (8)
13	10 (12)	8 (9)
14	14 (17)	13 (15)
15	12 (15)	13 (15)
Pupils (%)		
Both reactive	77 (94)	79 (92)
One reactive	3 (4)	3 (3)
Both unreactive	2 (2)	4 (5)
Volume of largest hematoma (mL)	25 (18–37) 11–96 Mean=31 (18.0)	23 (15–32) 10–97 Mean=27 (16.8)
Location of largest hemorrhage (%)		
Frontal	36 (44)	43 (50)
Temporal	39 (48)	37 (43)
Parietal	4 (5)	5 (6)
Occipital	3 (4)	1 (1)
Second hematoma present (%)	28 (34)	33 (38)
Time to randomization (h)	21 (13–31) 3–48 Mean=22 (11.7)	22 (14–28) 4–48 Mean=22 (10.6)

For continuous variables, median (quartiles) and range are presented plus mean and SD; for categorical variables, the number of cases (percentage) is presented.

IQR, interquartile range; SD, standard deviation; GCS, Glasgow Coma Score.

Of the 82 patients in the early surgery group, only 61 (74%) had surgery, 57 (93%) of these within 12 h of randomisation (see Table 2). The reasons for not having surgery were patient or relative refusal (15), improvement (1), deterioration (2), seizures (1), anesthetic risk (1), and change of history suggesting SICH rather than TICH (1). Although informed consent was obtained before randomization, patients often had more than one relative and further discussion could lead to a change of opinion. Of the 15 patients/relatives who refused surgery, 9 were in China and 5 in India. Of the 86 patients randomized to initial conservative treatment, 31 (36%) had surgery within 14 days of randomization, 10 (32%) of these within 12 h. The reasons for having surgery were neurological deterioration (29), no shrinkage in hematoma size (1), and rise in ICP (1). Neurological deterioration was identified by a drop in GCS, enlargement of the hematoma or increase in mid-line shift, increase in weakness, or change in pupil size or reactivity.

**Table 2.** Surgery Details for Early Surgery Patients Who Had Surgery and Initial Conservative Patients Who Required Delayed Surgery

	*Early surgery surgical cases* (N*=61; 74%)*	*Initial conservative treatment surgical cases (*N*=31; 36%)*
Method (%)		
Craniotomy	59 (97)	25 (81)
Other	2 (3)	6 (19)
Bone flap replaced (%)	47 (77)	13 (42)
Other cranial surgery (%)	1 (2)	3 (10)
Paralyzed and sedated (%)	17 (28)	12 (39)
Any noncranial surgery (%)	1 (2)	2 (7)
Preoperative GCS–eye (%)		
1	5 (8)	15 (48)
2	18 (30)	8 (26)
3	19 (31)	5 (16)
4	19 (31)	3 (10)
Preoperative GCS–Verbal (%)		
1	13 (21)	16 (52)
2	15 (25)	7 (23)
3	6 (10)	5 (16)
4	18 (30)	0 (0)
5	9 (15)	3 (10)
Preoperative GCS–Motor (%)		
1	0 (0)	4 (13)
2	2 (3)	1 (3)
3	6 (10)	3 (10)
4	4 (7)	6 (19)
5	26 (43)	14 (45)
6	23 (38)	3 (10)
Time randomisation to surgery (h)	3 (1–6) <1–24 Mean: 4 (4.5)	25 (6–79) <1–318 Mean: 58 (75.6)
Surgery within 12 h of randomization (%)	57 (93)	10 (32)
Time injury to surgery (h)	23 (16–36) 4–69 Mean 26 (13.8)	45 (26–99) 9–332 Mean 78 (79.0)
Surgery within 12 hours of injury (%)	9 (15)	3 (10)

For continuous variables, median (quartiles) and range are presented plus mean and standard deviation; for categorical variables, number of cases (percentage) are presented.

GCS, Glasgow Coma Score.

Surgical patients in the early surgery group were more likely to have craniotomy than those in the initial conservative group (97% vs. 81%; *p*=0.016, Fisher's test). One patient in the initial conservative group had burrhole surgery, but all others who did not have craniotomy had craniectomy. The bone flap was more likely to be replaced in the surgical patients in the early surgery group (77%) than in the initial conservative group (42%; Fisher test's, *p*=0.001). As Table 2 demonstrates, surgical patients in the early surgery group had significantly higher preoperative GCS scores for all subscales than those requiring surgery in the initial conservative group. Comparison of the baseline characteristics of patients in the initial conservative group who had surgery, with those that did not, showed that patients who deteriorated and went on to have surgery had larger hematomas initially (Mann-Whitney's test, *p*=0.010) and were more likely to have at least one pupil unreactive (Fisher's test, *p*=0.0005), but did not differ on age, GCS *at the time* of randomization, or presence of a second hematoma.

At 2 weeks postrandomization, there were similar proportions of patients in the two groups who were still on the neurosurgical ward: 29 (35%) of the early surgery patients and 32 (37%) of the initial conservative patients. Further, similar proportions had been transferred to another ward or hospital (3 [4%] and 4 [5%], respectively). However, 43 early surgery patients (52%) had been discharged, compared to 33 of the initial conservative patients (38%). Further, there was a significant difference in the percentage that had died by 2 weeks: 7 (9%) early surgery patients compared to 17 (20%) initial conservative patients (Fisher's test, *p*=0.047). At some point in the first 2 weeks, 7 (9%) early surgery patients were ICP monitored compared to 16 (19%) initial conservative patients (*p*=0.073), and this affected management decisions in 1 early surgery patient compared to 10 initial conservative patients (*p*=0.069). Patients were less likely to be monitored in India, where the ICP monitoring rate was 4% (3 of 74), compared to 21% elsewhere (20 of 94). Very few postrandomization events were recorded during the first 2 weeks of the hospital stay: Pneumonia was reported in 8 early surgery patients and 8 initial conservative patients, ischemic stroke (0 and 1), pulmonary embolism (1 and 2), postoperative extradural (0 and 2), septicemia (1 and 0), urinary tract infection (1 and 0), seizures (3 and 0), and other (5 and 1).

### Primary outcome

**FIG. 2.**
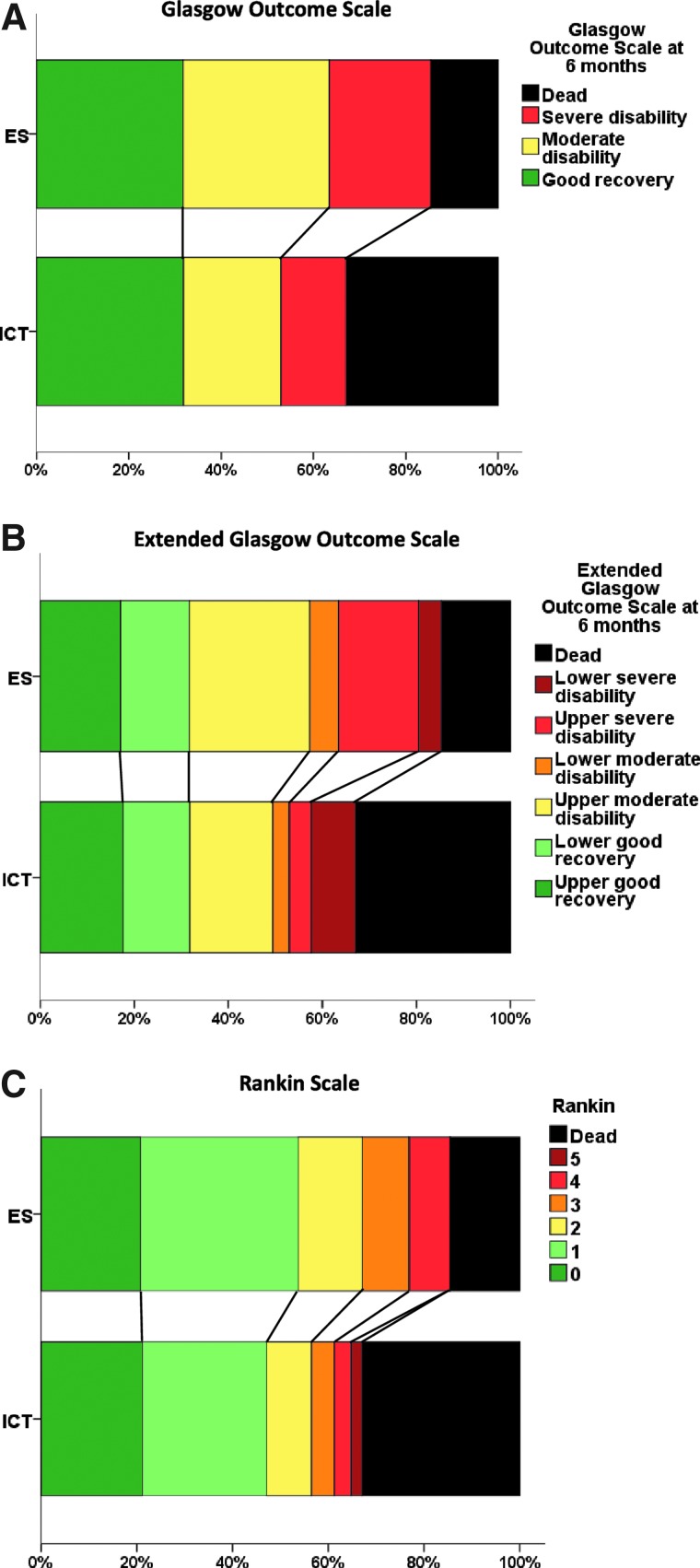
Outcome at 6 months. Statistical significance tests for outcome. (**A**) Proportional odds model, *p*=0.153; chi-squared for trend, *p*=0.047; outcome. (**B**) Proportional odds model, *p*=0.127; chi-squared for trend, *p*=0.052; outcome. (**C**) Proportional odds model, *p*=0.147; chi-squared for trend, *p*=0.043. ES, early surgery; ICT, initial conservative treatment.

Six-month outcome was available for 82 early surgery patients and 85 initial conservative patients; 1 patient from the initial conservative group was lost to follow-up. Fifty-two (63%) early surgery patients had a favorable outcome on the dichotomized GOS, compared to 45 (53%) initial conservative patients (OR, 0.65; 95% CI, 0.35, 1.21; *p*=0.171); an absolute difference of 10.5% (95% CI, −4.4, 25.3; see table 3; Fig. 2). Adjusting for age, volume, and GCS gives an OR of 0.58 (95% CI, 0.29, 1.16; *p*=0.122).

**Table 3.** Outcomes at 6 Months

	*Early surgery*	*Initial conservative treatment*	*Test and* p *value Absolute difference (95% CI)*	
Primary outcome (%)	*N* = 82	*N* = 85		
Unfavorable	30 (37)	40 (47)	χ^2^	*p* = 0.170
Favorable	52 (63)	45 (53)	10.5 (−4.4–25.3)	
Secondary outcomes	*N* = 82	*N* = 85		
Mortality at 6 months (%)				
Dead	12 (15)	28 (33)	χ^2^	*p* = 0.006
Alive	70 (85)	57 (67)	18.3 (5.7–30.9)	
Rankin (%)				
Unfavorable	27 (33)	37 (44)	χ^2^	*p* = 0.159
Favorable	55 (67)	48 (56)	10.6 (−4.0–25.3)	
GOS (%)				
Dead	12 (15)	28 (33)	χ2 trend	*p* = 0.047
Vegetative	0 (0)	0 (0)		
Severely dependent	18 (22)	12 (14)	POM	*p* = 0.153
Moderately dependent	26 (32)	18 (21)		
Good recovery	26 (32)	27(32)		
GOSE (%)				
Dead	12 (15)	28 (33)	χ^2^ trend	*p* = 0.052
Vegetative	0 (0)	0 (0)		
Lower SD	4 (5)	8 (9)	POM	*p* = 0.127
Upper SD	14 (17)	4 (5)		
Lower MD	5 (6)	3 (4)		
Upper MD	21 (26)	15 (18)		
Lower GR	12 (15)	12 (14)		
Upper GR	14 (17)	15 (18)		
Rankin (%)				
0	17 (21)	18 (21)	χ^2^ trend	*p* = 0.043
1	27 (33)	22 (26)		
2	11 (13)	8 (9)	POM	*p* = 0.147
3	8 (10)	4 (5)		
4	7 (9)	3 (4)		
5	0 (0)	2 (2)		
Dead	12 (15)	28 (33)		
EuroQoL Index				
Median	0.80	0.71	M-W	*p* = 0.218
Quartiles	0.52–1.00	0.00–1.00		
Range	−0.33–1.00	−0.59–1.00		
Limb movement (%)				
Worst affected leg^a^				
Unaffected	50 (72)	47 (82)	χ^2^	0.374
Weak	18 (26)	9 (16)		
Paralysed	1 (1)	1 (2)		
Worst affected arm^a^				
Unaffected	48 (70)	43 (75)	χ^2^	0.464
Weak	21 (30)	14 (25)		
Paralysed	0 (0)	0 (0)		

Number of cases (percentage) are presented; EuroQol utility index calculated using UK weightings provided by the EuroQol Group Foundation; tests conducted were χ^2^ (chi-squared), χ^2^ trend (chi-squared for trend), POM (proportional odds model), and M-W (Mann-Whitney). For each test, the *p* value is given. Absolute differences with 95% confidence intervals are presented for binary outcomes.

^a^ One patient did not provide information about their limb movements.

GOS, Glasgow Outcome Scale; GOSE, Glasgow Outcome Scale Extended; SD, severe disability; MD, moderate disability; GR, good recovery.

### Secondary outcomes

**FIG. 3.**
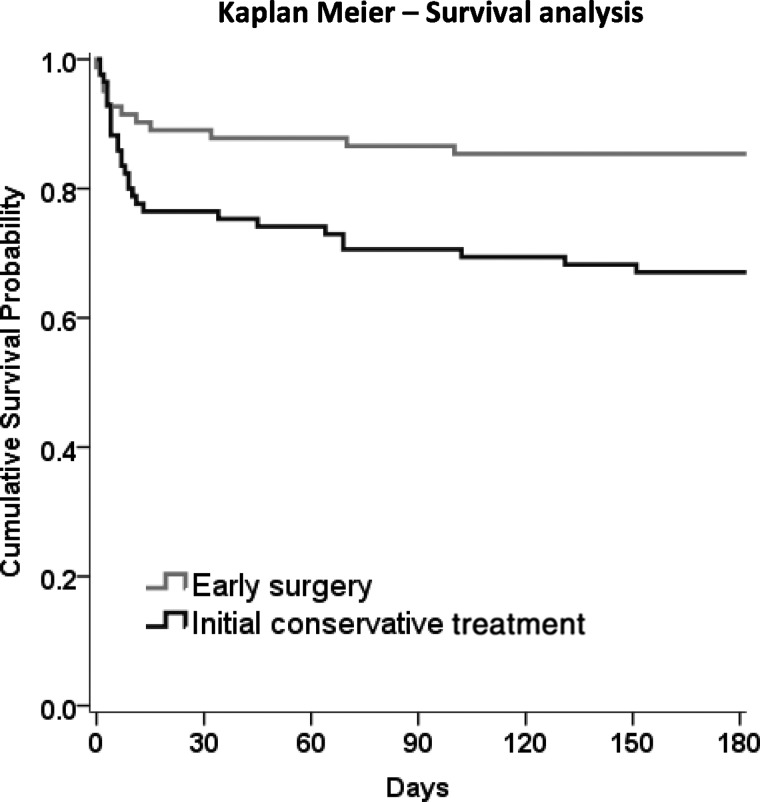
Kaplan-Meier's survival analysis. Log-rank test, *p*=0.0081.

However, there was a highly significant difference in mortality at 6 months, with 12 (15%) early surgery patients dying compared to 28 (33%) initial conservative patients (OR, 0.35; 95% CI, 0.16, 0.75; *p*=0.007), for an absolute difference of 18.3% (95% CI, 5.7, 30.9). Figure 3 shows the Kaplan-Meier's plot of survival for the two groups of patients, illustrating the significant survival advantage of early surgery compared with initial conservative treatment (*p*=0.008). Table 3 and Figure 2 show the distribution of GOS, GOSE, and Rankin at 6 months by treatment group. For each of these secondary outcomes, there is a significant trend in better outcome in the early surgery group (chi-squared trend: *p*=0.047, *p*=0.052, and *p*=0.043 respectively), although the proportional odds models did not reach statistical significance (OR, 0.67; 95% CI, 0.39, 1.16; *p*=0.153; OR, 0.66, 95% CI, 0.38, 1.13; *p*=0.127; OR, 0.67; 95% CI, 0.39, 1.15; *p*=0.147).

The main causes of death were the initial head injury (5 early surgery, 14 initial conservative) and pneumonia (4 early surgery, 2 initial conservative). Other causes in the initial conservative treatment group included cachexia (2), ischemic stroke (2), meningitis (1), pulmonary embolism (2), renal (1), TBI and surgery (1), seizure (1), and unknown– sudden death in the community (1). In the early surgery group, the other causes were hypovolemic shock (1), pulmonary embolism (1), TBI and surgery (1), and unknown in the community (1). Only 8 non-death-related major AEs were recorded: seizure (3); new/enlarged hematoma (2); infection (2); and other (1).

**FIG. 4.**
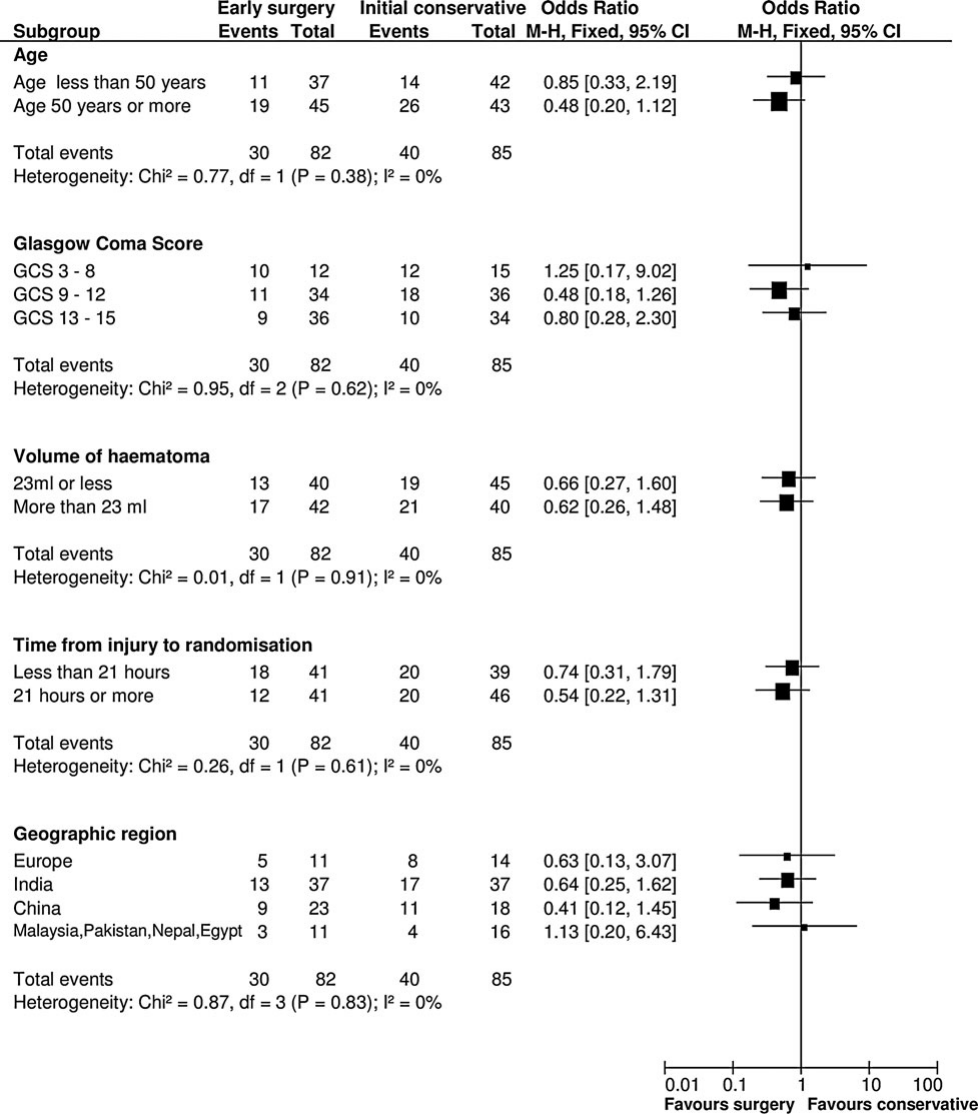
Subgroup analysis. M-H, Mann-Whitney; CI, confidence interval; GCS, Glasgow Coma Score.

Prespecified subgroup analyses are shown in Figure 4. None of the subgroups displayed any significant heterogeneity of treatment response, although the patients with a GCS of 9–12 had the best response from early surgery.

Among the patients who were allocated to early surgery and had surgery, 33% (20 of 61) died or were severely disabled at 6 months. However, 65% (20 of 31) of patients who were allocated to initial conservative treatment and had delayed surgery died or were severely disabled at 6 months, whereas 37% (20 of 54) of the conservative patients who did not have surgery had an unfavorable outcome.

An unadjusted comparison of raw mean costs showed that early surgery was, on average, $476 more costly than conservative management (Table 4). GLM regression analysis, adjusting for patient characteristics, showed early surgery to be $1,774 more costly (95% CI, −$284, $3,831) than initial conservative treatment. Sensitivity analyses showed that overall conclusions were robust to the choice of regression model for the analysis. Results from subgroup analyses were highly uncertain based on small sample sizes (and too small to conduct regression analysis) and should therefore be interpreted with caution.

**Table 4.** Costing Analysis (All Countries) and by Country Income Group

	*Early surgery (*N*=82)*		*Initial conservative treatment (*N*=86)*		
*All countries*	*Resource use; mean (SD)*	*Costs; mean (SD)*	*Resource use; mean (SD)*	*Costs; mean (SD)*	*Difference of means*
Cost surgery		981 (1678)		515 (1206)	
Cost ICU	4.18 (4.2)	2808 (5762)	4.06 (4.61)	2988 (6131)	
Cost HDU	1.72 (2.55)	385 (1053)	1.76 (3.01)	461 (1445)	
Cost ward	11.88 (15.95)	3595 (10,206)	14.24 (29.43)	3997 (13,789)	
Cost readmission	4.23 (14.43)	1145 (5775)	2.42 (9.63)	421 (1720)	
Total cost		8812 (18,032)^a^		8336 (18,685)^a^	+476 GLM model +1774 (95% CI, −284–3831)

*Low-income countries*	*Early surgery (*N*=6)*		*Initial conservative treatment (*N*=10)*		*Difference of means*
Cost surgery		142 (0)		14 (45)	
Cost ICU	0.83 (1.60)	203 (391)	1.2 (2.7)	293 (659)	
Cost HDU	3.83 (0.75)	468 (92)	3.5 (2.12)	427 (259)	
Cost ward	5.33 (1.03)	325 (63)	6.3 (6.43)	384 (392)	
Cost readmission	0 (0)	0 (0)	0 (0)	0 (0)	
Total cost		1139 (418)		1118 (614)	+20

*Low-middle-income countries*	*Early surgery (*N*=40)*		*Initial conservative treatment (*N*=39)*		*Difference of means*
Cost surgery		439 (511)		176 (369)	
Cost ICU	3.2 (3.78)	580 (1449)	2.38 (3.39)	227 (314)	
Cost HDU	1.93 (2.84)	87 (128)	1.97 (3.08)	168 (513)	
Cost ward	5.7 (5.84)	64 (98)	6.31 (5.29)	125 (364)	
Cost readmission	0.35 (2.21)	3 (20)	0.13 (0.59)	1 (5)	
Total cost		1174 (1583)		697 (964)	+477

*Upper-middle-income countries*	*Early surgery (*N*=28)*		*Initial conservative treatment (*N*=30)*		*Difference of means*
Cost surgery		1089 (1174)		822 (1031)	
Cost ICU	5.43 (3.79)	4272 (4134)	6.93 (4.49)	6010 (5588)	
Cost HDU	0.93 (1.84)	643 (1261)	1.17 (3.26)	821 (2295)	
Cost ward	16.86 (16.30)	3603 (4132)	14.27 (26.05)	3080 (6267)	
Cost readmission	8.39 (20.55)	997 (2986)	6.23 (15.48)	805 (1881)	
Total cost		10,603 (7517)		11,538 (10,149)	−936

*High-income countries*	*Early surgery (*N*=8)*		*Initial conservative treatment (*N*=7)*		*Difference of means*
Cost surgery		4927 (3617)		2020 (3542)	
Cost ICU	7.25 (5.95)	13,432 (14,847)	5.14 (6.72)	10,310 (15,989)	
Cost HDU	1.88 (3.18)	1089 (2668)	0.57 (0.98)	622 (964)	
Cost ward	30.25 (31.45)	23,671 (24,462)	69.71 (68.18)	34,662 (35,806)	
Cost readmission	12.27 (22.53)	8233 (16,895)	2.29 (6.05)	1719 (4547)	
Total cost		46,489 (38,880)^a^		47,483 (46,221)^a^	−994

^a^ Total mean cost is not equal to the sum of the resource use. This is because of the use of DRG costs per episode of care, applied to resource use in Germany.

SD, standard deviation; ICU, intensive care unti; HDU, high dependency unit; GLM, generalized linear regression model; CI, confidence interval.

## Discussion

Although the trial was stopped early by the UK NIHR-HTA with an associated reduction in statistical power, there were some clinically significant results. These included a statistically significant survival advantage (85% vs. 67%) and a nonsignificant benefit on GOS, both associated with early surgery.

Early management of patients with TICH is not harmonized around the world. Timing of surgery in patients with parenchymal hematomas post-TBI has not been standardized. This contrasts with patients who develop EDHs or acute SDHs, because guidelines (NICE 2nd edition) based on strong observational data^8,9^ have recommended early and expeditious scanning and surgery. Not all TICHs require removal and neither do all the contusions associated with them. Generally, clinical deterioration and expansion of the hematomas and their associated edema tend to trigger the need for surgery. If it were possible to anticipate these changes, then secondary brain damage would be avoided. The objective of the STITCH(TRAUMA) trial was to discover whether early surgery would prevent the secondary deterioration so often observed with conservative treatment. Though the primary outcome is not statistically significant, there is a strong signal that early surgery will indeed prevent such deterioration and save lives. This is noted in the highly significant reduction in mortality and the better outcomes in the ordinal analysis of the GOS and Rankin scales. A larger trial is urgently needed to confirm or refute this signal, which is particularly strong in patients with a randomization GCS of 9–12. This same group of patients with SICH had the best outcomes with early surgery in a previous study.^23^

Some units in some countries routinely measure ICP whereas others do not. In this study, 86% of patients were not monitored for ICP either because the hospital did not have the technology available or because they do not routinely use it for this patient group.^24^ The question of early surgery for TICH may be particularly important in those countries that do not measure ICP. An analysis of global ICP utilization trends and aspirations has confirmed that many countries in the world do *not* have the facilities and resources for ICP monitoring.^25^ It is in these very countries that this STITCH trial is most relevant to the question: Should early surgery be undertaken in patients with TICH when there is no option for ICP measurement?

There were crossovers from initial conservative treatment to early surgery and vice versa, as occurs in all surgical trials. This is because surgeons feel compelled to provide rescue surgery to those patients randomized to initial conservative treatment who subsequently deteriorate. On the other hand, some patients who were randomized to early surgery did not have surgery because their families withdrew consent. Despite these crossovers, the absolute benefit of early surgery exceeded 10% and was almost statistically significant. If the total of 840 planned patients had been recruited and if the same trend had transpired, this would have been a statistically significant result. In addition, the patients who had delayed surgery had deteriorated to a much poorer clinical state and this was associated with a much poorer outcome (65% dead or severely disabled, compared to only 33% in those operated upon early). This observation supports the primary hypothesis that early surgery is advantageous for TICH.

An analysis of outcome by whether patients actually had surgery or not is complex and biased because the decision for surgery was guided by the study in some cases and by a change in status in others: Some patients would have surgery before deterioration and others would only have surgery after deterioration. In the nonsurgery group, some patients did not have early surgery mainly because the relative refused, whereas others did not have surgery because that was the allocation (and either they did not deteriorate or if they did their surgeon or anesthetist did not decide to take them to surgery). We will publish a separate article that will address the driving forces behind the crossovers. We will also publish a separate description of the CT characteristics at baseline and at 5 days in those patients who survived.

Predicting which patients will deteriorate is complex, and the Surgical Trial in Intracerebral Haemorrhage (STICH II) identified a small number of patients (GCS between 9 and 12) that may benefit from such anticipatory treatment.^23^ In general, SICH patients with a good prognosis (GCS between 13 and 15) can be safely observed and only require craniotomy if they deteriorate. This is because there is enough time to perform a craniotomy before other secondary mechanisms, such as brain edema, mass effect with herniation, and reduced CPP from elevated ICP, cause harm. This may also be true for TICH patients. In particular, those TICH patients with an initial GCS between 9 and 12 had the best outcome with early surgery. The economic analysis indicates that a strategy of early surgery is associated with a nonsignificant increase in health care costs. Further work will estimate the cost-effectiveness of early surgery versus conservative management using 6-month and 1-year follow-up data. This will demonstrate the magnitude of additional costs over the longer term, as well as whether any additional costs are associated with sufficient benefits in terms of improvements in quality of life, measured by the EQ-5D, and length of life.

## Conclusion

A larger trial is needed to confirm this potentially very beneficial effect of earlier surgery. In the interim, there is a strong case for operating on patients with TICH who have a GCS of 9–12. Those who are alert or just confused (GCS 13–15) can probably be watched carefully for any deterioration because there is a safety margin, which diminishes the lower down the GCS the patient descends. Once the GCS has descended below 9, surgical intervention appears to be less effective. A strategy of early surgery is associated with a small, nonsignificant increase in health care costs, but further analysis using longer-term follow-up data are required to establish better estimates of costs and cost-effectiveness.

## Appendix

### STITCH (TRAUMA) Investigators

Principal investigators: A. David Mendelow, MB, BCh, PhD, FRCS; Barbara A. Gregson, BSc, PhD, FSS; Patrick M. Mitchell, BA, MB, BChir, BSc, FRCS, PhD; Andy Unterberg, MD, PhD; Elaine M. McColl, BA, MSc, PhD; Iain R. Chambers, BSc, PhD, CEng, FIPEM; Paul McNamee, MA, MSc, PhD.

Steering committee: Mr. J. Steers (independent chairman); Mr. Andy Vail (statistician); Dr. D. Birchall (neuroradiologist, independent member); Mr. Jake Timothy (neurosurgeon, independent member); Professor Luke Vale (health economist, independent member); Mr. A. White (lay member, Headway); Mr. D. O'Meara (lay member, UKABIF); Professor A.D. Mendelow; Dr. B.A. Gregson; Mr. P.M. Mitchell; Dr. A. Unterberg; Professor E.M. McColl; Dr. I.R. Chambers; Professor P. McNamee; Dr. E.N. Rowan; Dr. D. Boyers; Dr. R. Francis.

Data monitoring committee: Professor P. Hutchinson (chairman and neurosurgeon); Professor G.D. Murray (independent statistician); Dr. A. Gholkar (neuroradiologist).

Trial management team: Professor A. David Mendelow (chief investigator); Dr. Barbara A. Gregson (principal research associate and trial director); Dr. Elise Rowan (senior research associate and trial manager); Dr. Richard Francis (research associate and data manager); Dr. Dwayne Boyers (research associate and economist); Miss Helen Atkinson (trial administrator 2009–2010); Miss Courtenay Howe (trial administrator 2010–2013); Mr. Patrick Mitchell (neurosurgeon).

Radiological committee: A. Hassani; Y.K. Yap; L. Yap; A. Gholkar.

Writing committee: A.D. Mendelow; B.A. Gregson; E.N. Rowan; R. Francis; E. McColl; P. McNamee; I.R. Chambers; A. Unterberg; D. Boyers; P.M. Mitchell.

### List of Center Investigators by Country and Center Together with the Number of Patients Recruited (In this list, centers that recorded zero patients did return screening information)

Canada: Toronto, St Michael's Hospital (0): R.L. Macdonald.

Bulgaria: Sofia, University Hospital Pirogov (2): N. Gabrovsky; N. Velinov.

Czech Republic: Brno, University Hospital Brno (1): M. Smrčka; G. Hanoun.

Egypt: Alexandria, Alexandria University Hospitals (3): O.S. Abdelaziz; I.H. Zidan.

Germany: Heidelberg, Heidelberg University Hospital (2): A.W. Unterberg; C. Beynon; Munich, Bogenhausen Academic Teaching Hospital, Technical University of Munich (1): C.B. Lumenta; D.B. Schul; Ulm, University of Ulm School of Medicine (0): M.-E. Halatsch; A. Pala.

Hungary: Szeged, University of Szeged.

Neurosurgery (0): P. Barzo; B. Fulop.

India: Bangalore, BGS Global Hospital (1): S.A.V. Rao; N.K. Venkataramanaa; Bangalore, NIMHANS (5): S. Somanna; K.V.L.N. Rao; J. lal Gangadharan; Calcutta, AMRI Hospitals (0): R.N. Bhattacharya; Chennai, Fortis Malar Hospital (1): K. Sridhar; G. Venkatprasanna; Dehradun, Himalayan Institute of Medical Sciences (13): K.K. Bansal; C. Gupta, R. Kumar; Lucknow, King George's Medical University (erstwhile CSM Medical University) (29): S.K. Singh; C. Srivastava; B.K. Ojha; A. Chandra; Ludhiana, Christian Medical College & Hospital (3): S.S. Grewal; B. Gupta; Maharashtra, Acharya Vinoba Bhave Rural Hospital (3): A. Agrawal; Mullana (Ambala), MM Institute of Medical Sciences and Research (1): A. Agrawal; Mysore, Mysore Clinisearch (2): A. Sangli; New Delhi, All India Institute of Medical Sciences (8): P. Sarat Chandra; B.S. Sharma; Visakhapatnam, Care Hospital (8): P.V. Ramana; P.M. Jagannath.

Latvia: Riga, Pauls Stradins Clinical University Hospital (0): E. Valeinis.

Lithuania: Kaunas, Kaunas University of Health Sciences Hospital (2): A. Tamasauskas, R. Vilcinis; Klaipeda, Klaipeda University Hospital (0): A. Gvazdaitis.

Malaysia: Malaysia, Johor Bahru, Hospital Sultanah Aminah Johor Bahru (1): N.A.A. Rahman A. Ali; Kota Bharu, Kelantan, Universiti Sains Malaysia (6): J.M. Abdullah; T.Y. Chin.

Nepal: Biratnagar, Neuro Hospital (16): Y.B. Roka; P.R. Puri.

Pakistan: Peshawar, Northwest General Hospital & Research Center Peshawar (2): T. Khan; F. Filza.

People's Republic of China: Beijing, Tiantan Hospital affiliated to Capital Medical University (30): J. Zhao; L. Xu; J. Li; Shanghai, Huashan Hospital, Fudan University (9): Y. Sun; J. Hu; Tianjin, Tianjin Medical University General Hospital (4): S. Yang; R. Jiang.

Romania: Cluj-Napoca, Cluj County Emergency Hospital (1): I.S. Florian; M. Rus; Timisoara, Emergency County Hospital Timisoara (7): H. Ples; S.M. Marius.

Spain: Santander, University Hospital Marqués de Valdecilla (2): A. Vázquez-Barquero; Valladolid, Universitario Río Hortega (1): R. Sarabia; I. Arrese.

United Kingdom: Cambridge, Addenbrooke's Hospital, Cambridge University Hospitals NHS Foundation Trust (0): P.J. Kirkpatrick; A.G. Kolias; Dundee, Ninewells Hospital and Medical School (2): S. Eljamel; Haywards Heath, Hurstwood Park Neuroscience Centre (0): G. Critchley; J. Norris; Newcastle, Royal Victoria Infirmary (3): P. Bhattathiri; N. Ross; Southampton, Southampton General Hospital (1): A. Belli; D. Bulters.

United States of America: Los Angeles, Los Angeles County & University of Southern California Medical Center (0): J.P. Gruen; Philadelphia, Temple University Hospital (0): M.W. Weaver; F. Sultan; Portland, Legacy Emanuel Medical Center (0): J.W. Chen; S. Staat.
